# How the communicative development inventories can contribute to clinical assessments of children with speech and language disorders

**DOI:** 10.3389/fpsyg.2023.1176028

**Published:** 2023-07-14

**Authors:** Mårten Eriksson, Karin Myrberg

**Affiliations:** ^1^Faculty of Health and Occupational Studies, Department of Occupational Health Sciences and Psychology, University of Gävle, Gävle, Sweden; ^2^Centre for Research and Development, Region Gävleborg/Uppsala University, Uppsala, Sweden; ^3^Faculty of Health and Occupational Studies, Department of Caring Sciences, University of Gävle, Gävle, Sweden

**Keywords:** child health, language disorder, MB-CDI, multilingual children, speech and language therapy, language assessment

## Abstract

**Introduction:**

The purpose of the present study was to investigate whether information from the Swedish version of the Communicative Development Inventories III (SCDI-III) is informative to the Speech and Language Pathologist (SLP) when examining children with suspected speech and language disorders at a SLP unit.

**Method:**

Parents to 50 children (25 girls, 25 boys, age 30–80 months) that had been referred to the local SLP unit completed the SCDI-III. Nine children came from multilingual families and 41 children came from monolingual, Swedish speaking homes. The children were diagnosed as having developmental speech disorders (12) or developmental language disorders (33). Five children were not diagnosed with any disorder.

**Results:**

The results showed that the referred children performed significantly lower on scales for word production, grammar, and metalinguistic awareness, compared to a subset from the norms with a similar age and gender composition. Most children fell below the 10th percentile on word production and grammatical constructions. The intercorrelation between the three scales were in general substantial. Comparisons of children’s performance on the vocabulary and grammar scales of SCDI-III, and the medical records revealed 18 cases of discordance that would have motivated further examination. The parents rated sometimes their child’s vocabulary and grammar skills as higher and sometime as lower to the medical records.

**Discussion:**

Limitations due to attrition and sample size were discussed. It was concluded that the SCDI-III can provide valuable information to the examination at the SLP clinic in addition to parent interviews, observations of children, and various tests, and that the potential for adapted versions would be particularly high for examinations of multilingual children.

## Introduction

The MacArthur-Bates Communicative Development Inventories (CDI, Dale et al., 1989; Fenson et al., 1993, 2007) and the Language Development Survey (LDS, Rescorla, 1989) revolutionized the study of child language three decades ago by showing that parents could give valid and reliable information on infant’s and toddler’s concurrent communication skills. New versions have developed from the original two designed for English American speaking infants and toddlers, including short versions and extensions for children up to 3–4 years (Fenson et al., 2007; Garcia et al., 2014; Eriksson, 2017; Tulviste et al., 2020; Cadime et al., 2021; Kas et al., 2022; Mokhtari et al., 2022; Stolt, 2023). In addition, different versions of the instruments have been adapted to over 100 different languages and dialects^1^ probably making the CDI to the most widespread instrument around the world assessing child language.

Because of its easy administration, parent reports have allowed for studies of communication skills of large groups of children, revealing that the variation in young children’s language skills is much larger than previously thought (Fenson et al., 1994). Ease of administration also facilitated the collection of data from atypical children spread over large areas, for example children with autism spectrum disorders (Veness et al., 2012; Miniscalco et al., 2014), children with Down syndrome (Caselli et al., 1998; Berglund et al., 2001) and preterm children (Pérez-Pereira et al., 2014; Schults and Tulviste, 2016; Pérez-Pereira and Cruz, 2018; Tulviste et al., 2020). As the internet has become an integrated part of most families’ daily lives, digital versions of the CDI have been popular, and samples now often include data from several thousand children (e.g., Simonsen et al., 2014 included data from over 6,500 Norwegian children). The CDI has also been shown to have a remarkable predictive capacity. Assessments of Danish children at the age of 2 years with CDI predict academic achievement 10 years later (Bleses et al., 2016).

Despite the confirmations of earlier results and the many new findings made in child language based on parental reports, there also seems to be some limitations to their use. For example, one application is to use CDI as index tests in early screening for language difficulties. Yet, most reviews of screening studies including CDI or LDS as index test indicate that no study present a diagnostic accuracy sufficiently high for general screening (Law and Roy, 2008; Siu and US Preventive Services Task Force, 2015; Eriksson, 2022). However, there are other clinical applications for which parental reports on children’s communication skills might be useful which are hitherto not fully researched.

Considering the increasing migration in the world, often due to wars or natural disasters, and the many adaptations of the CDI into different languages and dialects, the CDIs have a potential to provide a valuable source of information to Speech and Language Pathologists (SLPs) in addition to standard procedures when meeting a multilingual child at the SLP unit. The particular languages migrants speak tend to vary rather quickly which makes the task of assessing language status in children from migrant families even more challenging because there is little time to develop new instruments for the new languages. The great number of CDIs from different language communities is therefore a potential resource to be exploited.

The present study concerns how the CDI could be useful to a SLP in assessing whether a child being referred to a SLP unit has a language disorder or not. This should not be conflated with screening as the children visiting the clinic are already screened and found positive.

### The present study

The study was situated in Sweden where around one-third of all pre-school children are multilingual (Statistics Sweden, 2022). Most children, including children from migrant families, have regular contact with their local Child Health Clinic (CHC) during their first 5 years (Wallby and Hjern, 2011). At the CHCs, children’s development and health are checked following a predetermined schedule at ages 2:5–3:0, 4:0 and 5:0 years. The first and second check-ups includes screening for language difficulties.

The children were referred to the SLP unit because they had failed the first screening at the CHC. This screening test consisted of five questions assessing language comprehension and an observation whether the child combines words. It was introduced and validated for 3-year old children by Westerlund and Sundelin (2000) and is a standard routine in half of Sweden. It was recently modified and validated for children 2:5 years (Nayeb et al., 2019) and for bilingual children (Nayeb et al., 2021). Bilingual children are first screened in one language (Swedish or the mother tongue, the order may vary). If they pass the first time, no further action is taken. If they fail the first time, the children are screened at a second occasion in their other language. Most children in the present study were screened at 2:5 years.

At the SLP unit, the child’s expressive and receptive language were thoroughly examined using a combination of parent interview, informal assessments and formal assessments including various standardized tests in agreement with the description from the Catalise consortium (Bishop et al., 2016). The results from these examinations, which may be extended over several meetings, are documented by the SLP in the child’s medical record. Multilingual children are to demonstrate difficulties in all their languages to be diagnosed with a speech or language disorder (*cf.* Bishop et al., 2016).

Children from Syria and Somalia were included as examples of migrant children. Because no standardized language tests in Syrian Arabic or Somali are available at Swedish SLP units, examinations of multilingual children in these languages rely heavily on language samples and dynamic assessments using an interpreter. Based on the SLPs examination, children with disorders are identified and diagnosed according to the International Statistical Classification of Diseases and Related Health Problems, 10th edition (WHO, ICD-10, https://www.who.int/standards/classifications/classification-of-diseases). All Services from the CHCs and SLPs in Sweden are free of charge.

We have used the Swedish version of CDI-III, SCDI-III, which is normed for Swedish speaking children 30–48 months old (Eriksson, 2017) in the present study. Younger children than 30 months old are rarely assessed at the SLP units in Sweden, and there is no CDI for older children. The SCDI-III differs in some respects from the version first introduced by Fenson et al. (2007) and early adaptations to Spanish (Guiberson and Rodrígez, 2010; Guiberson et al., 2011) and Basque (Garcia et al., 2014). The differences include a vocabulary part restricted to four semantic categories with a focus on verbs and abstract nouns, see Eriksson, 2017 for details). This change was introduced to reduce ceiling effects, a problem that had afflicted the original version (Fenson et al., 2007). It draws upon earlier work on the composition of children’s early lexicon which have shown that an increase in verbs comes as a second wave after an initial increase in number of nouns in many languages (Bates et al., 1994; Caselli et al., 1995; Bornstein et al., 2004; Schults and Tulviste, 2016) including Swedish (Berglund and Eriksson, 1994). The focus on verbs increased the difficulty of the scale and presumably facilitated the reporting task for the parent as only words from four semantic categories had to be searched in memory. Indeed, no ceiling effects were found after this change in the vocabulary part of SCDI-III for 4-year-old Swedish speaking children (Eriksson, 2017) or for Estonian (T. Tulviste, personal communication December 2, 2022) nor in Norwegian 4-year-olds (E. Holm, personal communication December 2, 2022), two additional languages with the same modification in the vocabulary section of CDI-III as SCDI-III. Another novel feature of all SCDI-III scales (vocabulary, grammar and the child’s vocabulary metalinguistic awareness) was that data fitted best to a linear function in contrast to scales of vocabulary and grammar developed for younger children in both English and other languages including Swedish for which an exponential function gave the best fit.

Norms are central as they disclose a child’s communication skills compared to those of other same-aged children. However, norms are sometimes used as a proxy for identifying children with language difficulties, for example children below the 10th percentile (Fenson et al., 2007; Eriksson, 2022). Yet, the validity of such proxy’s is in general unsubstantiated, that is, there is a lack of studies showing that children performing below the 10th percentile are diagnosed with a language disorder by SLPs, and that children scoring above the 10th percentile have no language disorder. In the present study, we plot the vocabulary, grammar, and metalinguistic scores of SCDI-III from children referred to a SLP unit in relation to the 10th percentile to characterize the children’s language. However, we make no claim of the 10th percentile bearing a particular significance apart from being a convenient reference in these figures. Neither should results from the present study be taken as evidence for or against the validity of a demarcation between children with and without a disorder at the 10th percentile because it only includes children with suspected language disorders (and the sample is way to small). Additionally, norms are based on group values and clinical judgments concern individual children. Moreover, judgments on children’s language status should be based on studies of more than one aspect of children’s communicative skills because a language disorder may distort the order in which language skills develops in a particular child (Eriksson, 2022), and performance of isolated aspects of language is not necessarily associated with functional language ability in everyday life (Spaulding et al., 2013).

The greatest potential for clinical use of parental reports is probably in relation to assessment of children from multilingual families (Freeman and Scroeder, 2022) as standardized tests in non-Swedish languages are not available at Swedish SLP units and communication with the parent often go by an interpreter. Yet, using the instruments with multilingual families might encounter new challenges, including, but not exclusively associated to the adaptation or translation of the instruments to other languages. There might also be advantages to investigate the use of SCDI-III in relation to assessment of children from monolingual (Swedish speaking) families, for example, some children perform below their actual competence on standardized tests due to shyness or lack of concentration. Therefore, the present study includes both children from monolingual Swedish-speaking using SCDI-III and multilingual families using translated or adapted versions of the SCDI-III.

To conclude, the overall aim of the present study was to investigate whether information from SCDI-III is informative to the SLP when examining children with suspected speech and language disorders at a SLP unit. The study includes both monolingual Swedish speaking children and multilingual children (Syrian Arabic and Somali). The following four research questions were asked:

1. Have children with a verified developmental speech disorder (DSD) or a verified developmental language disorder (DLD) lower scores on the three scales of SCDI-III (vocabulary, grammar and metalinguistic awareness) in comparison to normative data from a sample of same aged typical developing children?
2. Are the correlations between vocabulary, grammar, and metalinguistic skills the same for children with DSD, children with DLD and typical developing children from the norming group?
3. Do children with a DSD/DLD score below the 10th percentile on the three SCDI-III scales?
4. Is the information from the SCDI-III important to the SLP when deciding on a diagnosis, and is there any difference in this respect between children from monolingual Swedish speaking families compared to children from multilingual families?

## Method

### Participants

All preschool children being referred to a local SLP unit were eligible for inclusion. A total of 123 instruments were distributed by the SLPs to visiting parents: 13 in Syrian Arabic, 8 in Somali and 102 in Swedish. Of these, 7 in Syrian Arabic, 2 in Somali and 41 in Swedish were returned, corresponding to a response rate of 41%. The completed forms were from 25 girls and 25 boys with a median age of 54 months (range 32–80 months). The distribution of age group and gender over language is shown in Table 1.

**Table 1:** Age group, language, gender, and presence of language disorders in children referred to a SLP unit.

Age group(months)	Swedish		Arabic		Somali		Total	Girls	Boys	Girls	Boys	Girls	Boys
*Children with speech disorders n = 12*							
30–36							
37–42							
43–48							
49–54		3					3
55–60	4						4
61–66	2						2
67–80	2	1					3
*Children with language disorders n = 33*							
30–36	1	3		1		1	6
37–42	2	4					6
43–48	1			2			3
49–54	2	1	1	1			5
55–60	1						1
61–66	3	2	1		1		7
67–80	1	3	1				5
*Children with no speech/language disorders n = 5*							
30–36							
37–42							
43–48	1	1					2
49–54		1					1
55–60		1					1
61–66	1						1
67–80							
Total	21	20	3	4	1	1	50

*N* = 50.

### Instrument

The Swedish Communicative Development Inventory III (SCDI-III, Eriksson, 2017) designed for children 2 years 6 months to 4 years was used in its original Swedish form and in two preliminary versions, Syrian Arabic and Somali, respectively. The SCDI-III starts with a general question about the child’s general level of communication, with six alternatives from “no words” to “long and complicated sentences” including examples. If a parent marked “no words,” no more questions were applicable, and the parent was asked to hand in the form. Next follows a vocabulary list of 100 words with a focus on verbs and adjectives divided into four semantic categories; food related words, body related words, mental words, and emotion words. In a third section, 18 examples of grammar and sentence complexity was assessed. A fourth section contained seven questions on metalinguistic awareness. A final question concerned pronunciation.

The Syrian Arabic form of SCDI-III was developed with assistance from K. Floccia and A.G.S. Abdelwahab at the university of Plymouth, UK, who recently has published adaptation of the Words Only (short form) based on CDI and designed for children 8 to 30 months in 17 Arabic dialects (Abdelwahab et al., 2021). The development of the Somali version of SCDI-III started with a translation from the Swedish version by a professional interpreter. This translation was then back translated to Swedish by two Somali speaking SLP students in cooperation with I. Lundeborg Hammarström at Linköping University, Sweden. The grammar section of SCDI-III was reduced and included only three items illustrating use of elaborated phrases complexity in Syrian Arabic and Somali (Question 17, item 5, 8, and 9) because of difficulties comparing grammar development across languages.

### Procedure

Data was collected between Jan 2020 and Dec 2021. The parents were asked to complete the instrument at home and put it in a pre-stamped envelope addressed to the first author at the University of Gävle. All forms were returned within a 30-day period after the assessment. Hence, the ordinary SLP made their evaluation of children’s language as usual, blind to the information from SCDI-III.

## Analyses

### Group level

Because SCDI-III was designed for children 30–48 months old, no child older than 48 months was included in the group comparisons. First, all children with a verified disorder were compared to the norming group. A second comparison was then carried out including only monolingual Swedish speaking children. The normative group included 1,134 children, but this group was reduced in each comparison to reflect the exact age and gender composition of the clinical groups (Table 1).

Differences between children with verified speech and language disorders and normative values on vocabulary, grammar and metalinguistic awareness were determined by one sample *t*-tests.

Associations between expressive vocabulary, grammar, and metalinguistic skills were investigated by Pearson correlations. Only Swedish speaking children were included in the correlational analysis because the long grammar scale was not included in the non-Swedish versions of SCDI-III. Significance of the difference between two correlations were determined by a Z-test (Soper, 2023). To control for substantial disassociations between expressive vocabulary and grammar in a few children that might distort the correlation on the group level, we looked for the number of children for which a parent had reported vocabulary skills in the lowest quartile together with grammar skills in the top quartile, or vice versa.

Performance in relation to the 10th percentile of the normative sample (Eriksson, 2017) were illustrated in figures depicting this reference as a solid line.

### Individual level

Medical records from the SLPs examination adjacent with the date of completion of the SCDI-III were obtained for each child and scrutinized by an experienced SLP (KM), not clinically involved with any of the participants. The notes filed under the record keywords “vocabulary” and “grammar” were examined with particular rigour alongside with the keyword summarizing the child’s overall communicative ability. This information was compared with the information contained in the completed SCDI-IIIs and were qualitatively categorized as concordant or discordant. Discordant cases were categorized as higher as or lower on the vocabulary scale and the grammar scale than would be expected from the information in the medical records. Conflicting information was required in order for a case to be categorized as discordant. The metalinguistic category was excluded since the standard SLP assessment does not yield adjacent information.

### Research ethics

All participating parents consented to have data from their children included in the project and contributed actively by completing the SCDI-III. All data was treated confidentially, and the project was approved by the local ethical committee (dnr 2019–02780).

## Results

A total of 33 children were diagnosed with a developmental language disorder, DLD (F80.1, F80.2, F80.8 W, R470D) including the seven children with Arabic and two children with Somali background. Another 12 children were diagnosed with a developmental speech disorder, DSD (F80.0; R48.2B). All children with DSD were over 4 years. The disorders were quite evenly distributed over girls and boys. Five children had no speech or language disorder see Table 1.

### The clinical groups performance compared to that of a norming group

To investigate if children with DLD scored lower than children from the norming sample on the three scales from SCDI-III assessing vocabulary, grammar and metalinguistic scales, the mean value from the 15 children with DLD were compared to those of a subset of typical developing children described in Eriksson (2017) reflecting the same age and gender composition. The same analyses were also carried out including only the 11 monolingual Swedish speaking children as the validity of the CDI-III versions in Somali and Syrian Arabic has not been properly established and might therefore yield somewhat unreliable data.

### Vocabulary

The mean number of words on SCDI-III for the 15 children diagnosed with DLD and 48 months or younger was 20.47 words (*sd* = 18.38) out of 100 words. This was significantly lower than the norming value of 61.05 words taken from the norming sample of SCDI-III (Eriksson, 2017), reflecting the same age and gender composition based on 395 children, *t* (14) = − −8.55, *p* < 0.001, *d* = −2.21, *CI* [−3,15, −1.24] as determined by a one-sample *t*-test.

Exclusion of the 4 children under 48 months with Somali or Syrian Arabic as best language yielded a very similar result. The mean number of words was 19.73 (*sd* = 19.73) as compared to a norming value of 60.70 words based on 278 children, *t* (10) = − −8.41, *p* < 0.001, *d* = −2.54, *CI* [−3,77, −1.28] determined by a one-sample *t*-test.

### Grammar

The original grammar scale in SCDI-III was developed for Swedish and contained 18 items with a maximum score of 36. The mean score for the 11 monolingual Swedish speaking children, diagnosed with DLD and 48 months or younger, was 4.27 (*sd* = 6.20) as compared to a norming value of 22.29 based on 278 children. This difference is significantly lower, *t*(10) = − −9.64, *p* < 0.001, *d* = −2.91, *CI* [−4,28, −1.51].

Because comparisons of grammar across languages can be extremely difficult, we tried out a short grammar scale containing only three items reflecting how elaborate utterances the child typically use, that would be easier to use across languages. An example in which the parent should indicate which of two forms was most representative for the child’s current speech, is “Turn on the light” or “Turn out the light so I can see.” The correlation between the short and the full item scale for all children in the norming group was *r* = 0.91, *p* < 0.001, *n* = 1,120 (and for a selection reflecting the age and gender composition of the current sample, *r* = 0.835, *p* < 0.01, *n* = 320). The correlation in the present group was *r* = 0.954, *p* < 0.001, *n* = 38. Thus, it seems that much of the information from the full grammar scale can be captured by this short 3-item scale.

The mean score for the children with DLD (including one Arabic and one Somali child) on this short scale was 0.83, which should be compared to a norming value of 4.10 from the norming group, *t* (11) = −6.11, *p* < 0.001, *d* = −1.764, CI [−2.670, −0.828] as determined by a one-sample *t*-test.

### Metalinguistic awareness

The mean score on the metalinguistic scale (maximum of 7) for the children diagnosed with DLD and 48 months old or younger was 1.93 (*sd* = 1.49). This was significantly lower than the norming value of 3.52 taken from the norming sample of SCDI-III (Eriksson, 2017) reflecting the same age and gender composition based on 359 children, *t* (13) = − −3.82, *p* = 0.002, *d* = −1.02, *CI* [−1.66, −0.36] as determined by a one-sample t-test.

Exclusion of the children with Somali or Syrian Arabic as best language yielded a very similar result, now with a mean of 2.09 (*sd* = 1.30) as compared to a norming value of 3.44 based on 278 children, *t* (10) = − −3.44, *p* = 0.006, *d* = −1.04, *CI* [−1,76, −0.28] as determined by a one-sample *t*-test. In sum, the children with a verified language disorder scored as a group significantly lower than a norming group with a similar age and gender composition.

### Interrelations between vocabulary, grammar and metalinguistic awareness

The correlations between vocabulary, grammar and metalinguistic awareness were high in the reference group. The correlations were also high for all the Swedish speaking children in the present group, see Table 2. Breaking the already small study group into subgroups is associated with great uncertainty. Yet, separate analyses of children with DSD, DLD, and no language disorder indicated that the associations are substantial for children with DLD and for children with no language disorder. However, another pattern with no or even negative associations between the three skills are indicated for children with DSD (Table 2). The difference in correlation between vocabulary and grammar for children with DSD and children with DLD was indeed significantly different, *z* = 2.508, *p* = 0.001, as was the difference in correlation between vocabulary and metalinguistic skills, *z* = 3.271, *p* < 0.001. To further investigate the associations between vocabulary and grammar on the individual level, we looked for the number of children for which a parent had reported vocabulary skills in the lowest quartile together on grammar skills in the top quartile, or vice versa. However, no such children were found. Hence, the low correlation between vocabulary and grammar was not caused by a few odd reports with large negative correlations.

**Table 2:** Correlations between scales measuring expressive vocabulary, grammar, and metalinguistic awareness in the Swedish speaking children referred to the SLP unit.

	M	SD	Vocabulary	Grammar
*….Children from the reference group, n = 1,035–1,104*					
Vocabulary	65.05	18.06	–	
Grammar	23.53	9.28	0.785**	–
Metalinguistic awareness	3.67	1.91	0.548**	0.537**
*All Swedish speaking children referred to the SLP unit, n = 41*					
Vocabulary	55.37	30.24	–	
Grammar	16.17	11.61	0.773**	–
Metalinguistic awareness	4.02	2.09	0.739**	0.597**
*Children with speech disorders, n = 12 (mean age 61 months)*					
Vocabulary	78.25	10.11	–	
Grammar	26.50	5.00	−0.202	–
Metalinguistic awareness	5.50	1.00	−0.121	−0.091
*Children with language disorders, n = 24 (mean age 74 months)*					
Vocabulary	39.54	29.46	–	
Grammar	9.33	9.75	0.661**	–
Metalinguistic awareness	3.29	2.03	0.794**	0.491*
*Children with no speech/language disorders, n = 5 (mean age 55 months)*					
Vocabulary	76.40	15.75	–	
Grammar	24.20	7.12	0.714	–
Metalinguistic awareness	4	2.74	0.359	0.756

*p < 0.05, **p < 0.01.

### Do children with a DSD/DLD score below the 10th percentile on the three SCDI-III scales?

**Figure 1:**
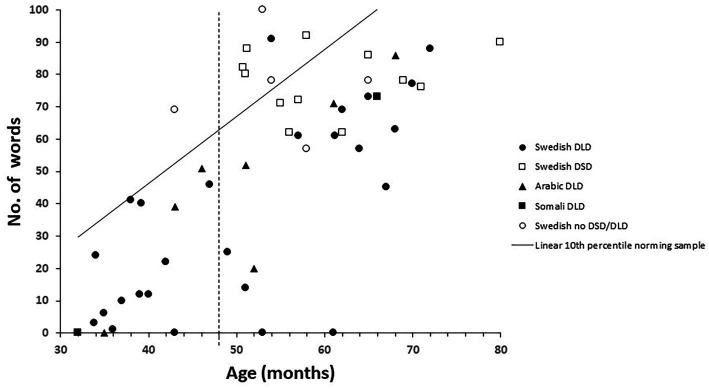
Vocabulary size plotted over age and related to the 10th percentile from the norming group in children with Developmental Language Disorders (DLD), Developmental Speech Disorders (DSD), and no disorder for children speaking Arabic, Somali, and Swedish.

**Figure 2:**
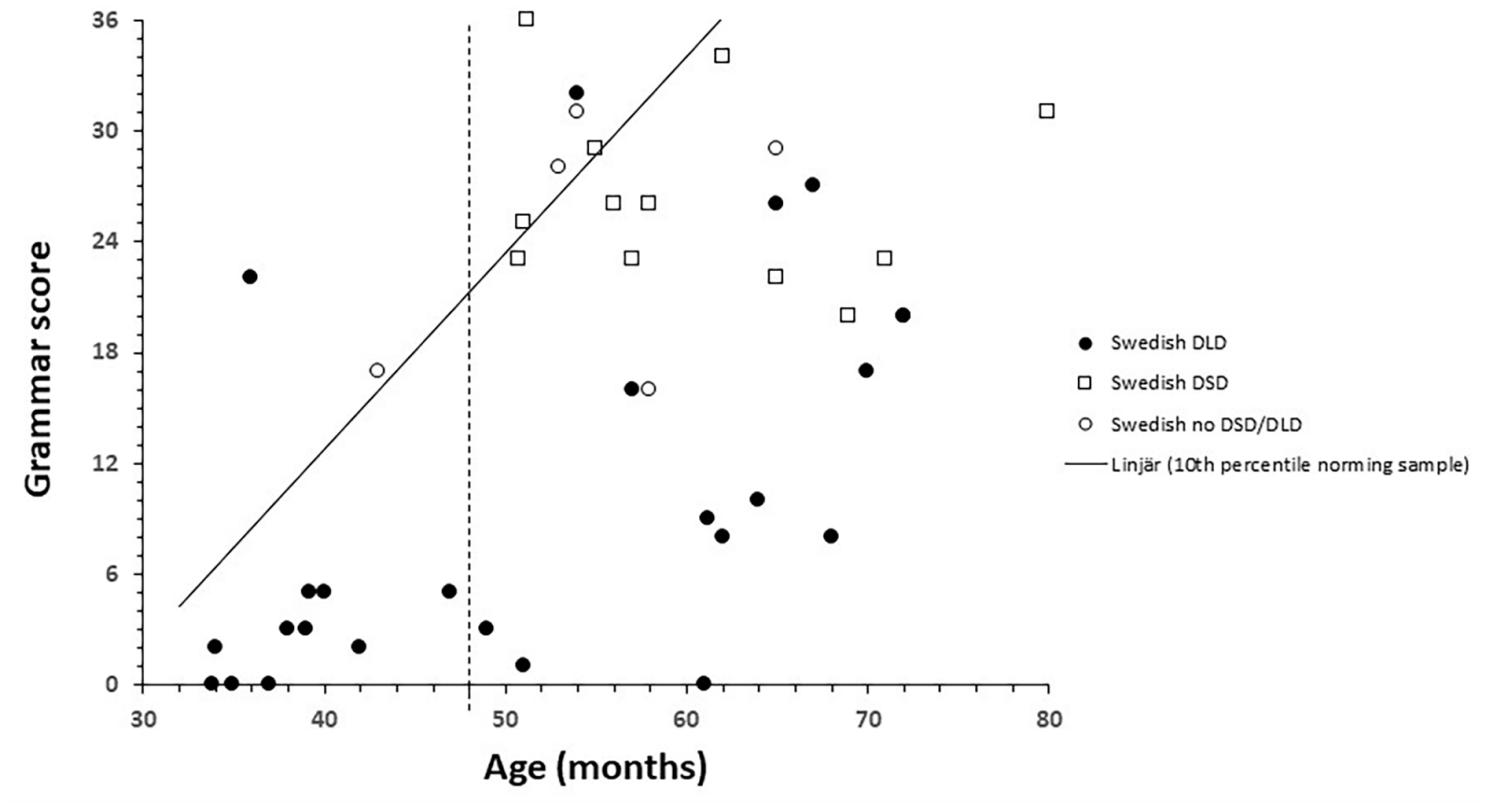
Grammar score plotted over age and related to the 10th percentile from the norming group in Swedish speaking children with Developmental Language Disorders (DLD), Developmental Speech Disorders (DSD), and no disorder.

**Figure 3:**
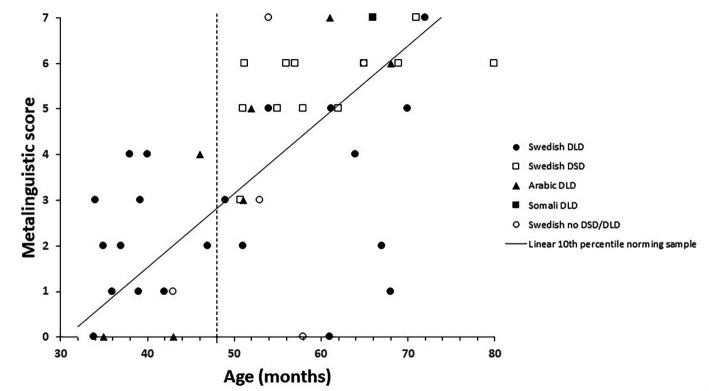
Metalinguistic score plotted over age and related to the 10th percentile from the norming group in children with Developmental Language Disorders (DLD), Developmental Speech Disorders (DSD), and no disorder for children speaking Arabic, Somali, and Swedish.

The 10th percentile from the norming sample is marked in Figures 1–3 by a solid line. The age of 48 months is marked with a vertical dotted line, and the 10th percentile to the right of this is thus an extrapolation. The 10th percentile distinguished perfectly between children with and without a language disorder 48 months or younger on the vocabulary scale. For the children 48 months or older with no speech or language disorder, two children scored below the 10th percentile on the vocabulary scale and among the children with DSD (all 48 months or older), eight out of 12 children scored below the 10th percentile, see Figure 1. All children, except one with DLD 48 months or younger, scored below the 10th percentile on the grammar scale. One child with DLD that were older than 48 months scored above the 10th percentile while two older children without DLD scored below the 10th percentile. Among the children with DSD, nine children out of 12 scored below on the grammar scale see Figure 2. On the meta-linguistic scale, slightly more than half of all children performed above the 10th percentile and three of the five children without a speech and language disorder scored below the 10th percentile see Figure 3.

In sum, the 10th percentile discriminated between children with and without a language disorder quite well on the vocabulary and the grammar scales while the 10th percentile on the metalinguistic scale was of little help in this respect. The discriminations were also best for children 48 months or younger. DSD differ from DLD in that the condition involves problems creating or forming speech sounds, not problems with expressive and/or receptive language. It was therefore surprising that a large proportion of children diagnosed with DSD were positioned below the 10th percentile on the scales.

### Is the information from the SCDI-III important to the slp when deciding on a diagnosis?

The issue of interest was whether the SCDI-III added new information to the assessment that motivated further investigation, or even presumptively could imply a revision of the child’s diagnosis. Comparisons of the children’s medical record information with the parental reports were made by qualitative analyses. We have listed the cases for which the SCDI-III provides information discordant with the medical record in Table 3. In 18 out of the 50 cases (36%), discordances were found. These cases were rather evenly distributed among gender, diagnostic group, and age span. Among the multilingual children, however, discordant results between the parent report and the medical record were observed in 6 out of 9 children (67%). An example of a discordance is child 13, diagnosed with DLD, for which the SLP reported an extremely limited expressive vocabulary and the parents report that she uses a rather large variety of the words. Here, the discordance was of the “higher” type indicating that the parents reported higher verbal skills than what was described by the SLP in the medical record. An example of the “lower” type of discordance is child 5, diagnosed with DSD, for which the parent reported much larger problems with vocabulary and grammar than was indicated in the medical records. In fact, all observed discordances including children with DSD were of this type. The review of the medical records indicates that vocabulary and grammar have been rather superficially examined in the children with DSD, with assessment primarily focusing on the production of speech sounds. The communicative skills in all six multilingual children were reported higher than assessed by the SLP (considering the child’s abilities across all languages). The Arabic and Somali SCDI-IIIs are based on the child’s abilities on their mother tongue. Although not formally assessed by a professional interpreter in all these cases, the medical records read that these children have none or very limited abilities on their mother tongue.

**Table 3:** Discordances between SLP assessment and SCDI-III.

Child	Age	Gender	Language	Vocabulary		Grammar		Months	Higher parent rating	Lower parent rating	Higher parent rating	Lower parent rating
*Children with speech disorders n = 4*							
5	71	Girl	Swedish		1		1
6	57	Girl	Swedish		1		1
31	62	Girl	Swedish		1		
39	70	Girl	Swedish		1		1
*Children with language disorders n = 13*							
13	57	Girl	Swedish	1		1	
15	35	Boy	Swedish		1		1
16	66	Girl	Swedish	1		1	
20	72	Boy	Swedish			1	
21	37	Boy	Swedish		1		1
22	61	Girl	Swedish	1		1	
37	54	Boy	Swedish	1		1	
41	50	Girl	Arabic	1		1	
42	68	Girl	Arabic	1		1	
44	46	Boy	Arabic	1		1	
45	53	Boy	Arabic			1	
47	43	Boy	Arabic	1		1	
49	67	Girl	Somali	1		1	
*Children with no speech/language disorders n = 1*							
50	58	Girl	Swedish		1		1
Total				9	7	11	6

*N* = 18.

## Discussion

In this study, parent reports in the form of SCDI-III were collected from children undergoing traditional examination at a SLP unit. Although the use of CDIs at SLP units are quite widespread, there is a scarcity of studies evaluating this practice. The present study yielded four findings; (1) the referred children scored significantly lower than previously established norms for scales measuring expressive vocabulary, grammatical constructions, and metalinguistic awareness, (2) most children performed below the 10th percentile on the two former scales, (3) the interrelations between scales were high with a possible exception of children with DSD, (4) discordances between the SCDI-III and information from the medical records were found in 36% of the children.

That the referred children scored considerably lower than norms reflecting typically developing children on SCDI-III was expected and suggest that SCDI-III is a highly relevant instrument for clinical use. The term “clinical validity” is sometimes used casually for such results (eg. Lopez et al., 2023), but the term was originally used to denote the classification accuracy of a screening test (Holtzman, 1999). Evidence for the latter meaning is not demonstrated in the present study because this is not a study on screening.

The intercorrelations between the scales measuring expressive vocabulary, grammar and metalinguistic awareness were generally high. This was also expected from previous research (eg. Fenson et al., 2007; Eriksson, 2017). The correlation between vocabulary and grammar is the best documented relation. Bates and Goodman (1997) argued that a high correlation between vocabulary and grammar indicate that they operate together, in contrast to linguistic theories placing them in different modules. Furthermore, they give evidence of a high correlation between vocabulary and grammar in quite diverse populations including typically developing children, children with focal brain injury, children with Williams syndrome, children with Down syndrome, aphasic patients, and studies of on-line language processing in healthy adults. In this light, the low correlation between vocabulary and grammar in the group of children with DSD is intriguing. Although the group with DSD was small (n = 12), the difference in correlation between vocabulary and grammar for this group and children and those with DLD was significant. The main differences between children with DSD and children with DLD is that children with DSD have problems in creating or forming speech sounds but, in contrast to children with DLD, not problems with expressive or receptive language. It can also be seen from Figures 1, 2, that the children with DSD score rather high in both vocabulary and grammar. A possible explanation to the low correlation between vocabulary and grammar might instead be related to the fact that problems in creating speech sounds is normal for toddlers, and the diagnosis DSD is therefore only given to older children. This restricts the variation in both vocabulary and grammar and the low correlation might therefore be a consequence of the low variation. More studies involving a larger group of children with DSD including a larger variation in vocabulary and grammar skills is needed to confirm this.

Complete agreement between the clinical examination and the SCDI-III would have made one of them redundant. However, discordance was fairly common, and the SCDI-III indicated sometimes that the child had more speech and language problems than recorded by the SLP, sometimes less. Thus, it was not the case that parents always rated their children’s communication skills as higher than the SLP. Discordance of the opposite sort was observed for four children with DSD. The review of the medical records of these children indicated that vocabulary and grammar was rather superficially examined, probably due to referral information concerning speech-sound problems. In such cases with very specific information in the referral, SCDI-III can provide convenient information on whether to expand the examination or not.

All nine multilingual children enrolled in this study were diagnosed with DLD and discordances between the SCDI-III results and the SLP were observed in six of them. Here, all six parents reported higher linguistic competence than assessed by the SLP. These conflicting results might reflect confounding factors associated with testing multilingual children. However, conflicting information of this kind is crucial to the SLP in deciding whether a more thorough examination of a child should be undertaken or not. It might also have motivated a change in the treatment plan or in the advice given to parents.

### Strengths and limitations

Notably, this study includes both strengths and limitations. One major strength is that the study employs the SCDI-III designed for children 30–48 months old to children being referred to a SLP unit. This age range corresponds better to the age of the referrals than what CDI versions designed for younger children do. In fact, many of the referrals were older than 48 months. Another strength of the study was that the SLPs were blind to the results from SCDI-III. Hence, they followed just standard procedures when examining the children and were not influenced by the child’s score on SCDI-III. This made it possible to study what SCDI-III possible could add to the standard procedure. A third strength was that the study took place in a community setting and the children had been subject to a professional SLP examination with associated ICD diagnoses.

A major limitation was that we lost control over the attrition during the pandemic. The original plan was to deliver SCDI-III consecutively to eligible children at their first visit to a SLP unit until information on 50 multilingual children (Syrian Arabic or Somali besides Swedish) were reached, and information from whatever the number of monolingual Swedish speaking children that was gathered in that time. However, priorities in the health care changed during the pandemic, and research on child language was not prioritized. Numerous appointments were redirected to telemedicine and information about the meeting was delivered by mail. Information on the current project was at first included in this information but dropped off gradually to restart again at the second half of 2021. It is also possible that attrition among the youngest children relates to a very limited expressive ability in many cases. A quick glance at the items in the questionnaire by the parent might have led to misassumptions about the target group being children with more advanced language than their child. Other reason to the attrition might reflect low parental interest in the study, little time available to complete the questionnaire, and low literacy levels in the parents. Contacts with multilingual families were also hampered by the need for an interpreter. Furthermore, a substantial attrition was associated to a small sample size. Future studies with larger study samples to validate these results, particularly for multilingual children, are warranted. To achieve this, it would probably be beneficial to ask parents to fill in the report during- or adjacent to the child’s visit to the SLP unit.

## Conclusion

Overall, the results indicate that the SCDI-III would be a useful instrument in addition to parent interviews, observations of children, and standardized tests in examinations of pre-school children at the SLP clinic. In particular, further assessments are warranted when the result from SCDI-III is discordant to other results in the examination process. The potential is probably higher for multilingual children than for monolingual children, but more research including more multilingual children, and also more languages, are needed. The clinical use of SCDI-III described here should in principle be the same for adaptations to other languages. It is therefore important that CDI-IIIs are developed for more languages, and that these versions can be made available to SLP around the world from one common website.

## Data availability statement

The raw data supporting the conclusions of this article will be made available by the authors, without undue reservation.

## Ethics statement

The studies involving human participants were reviewed and approved by Swedish Ethical Review Authority. Written informed consent to participate in this study was provided by the participants' legal guardian/next of kin.

## Author contributions

ME and KM contributed to the conceptualization, methodology, and analyses of the study. ME wrote the original draft. All authors contributed to the article and approved the submitted version.

## Funding

The research was supported by the University of Gävle, Sweden.

## Conflict of interest

The authors declare that the research was conducted in the absence of any commercial or financial relationships that could be construed as a potential conflict of interest.

## Editor’s note

Maria-José Ezeizabarrena edited the article in collaboration with Melita Kovacevic, University of Zagreb, Zagreb, Croatia.

## Publisher’s note

All claims expressed in this article are solely those of the authors and do not necessarily represent those of their affiliated organizations, or those of the publisher, the editors and the reviewers. Any product that may be evaluated in this article, or claim that may be made by its manufacturer, is not guaranteed or endorsed by the publisher.

## Footnotes

^1^ https://mb-cdi.stanford.edu
